# Corneal Melt and Perforation Associated With Prolonged Oral Non-Steroidal Anti-Inflammatory Drug (NSAID) Use: A Case Report

**DOI:** 10.7759/cureus.60853

**Published:** 2024-05-22

**Authors:** Reham Aljehani, Mohammed Almutlaq, Shaikha Aldossari, Khaled Al-Abduljabbar

**Affiliations:** 1 Ophthalmology, King Abdulaziz University, Jeddah, SAU; 2 Ophthalmology, Cornea and External Diseases, King Khaled Eye Specialist Hospital, Riyadh, SAU; 3 Ophthalmology, King Khaled Eye Specialist Hospital, Riyadh, SAU

**Keywords:** cornea abnormalities, nsaid abuse, (nsaid) non-steroidal anti-inflammatory drugs, corneal wound healing, corneal thinning, corneal perforation

## Abstract

Corneal melt and perforation can arise from various etiologies, including the use of toxic topical drops, particularly topical non-steroidal anti-inflammatory drugs (NSAIDs). The literature has frequently documented the association between the use of topical NSAIDs and the subsequent development of corneal ulcers. More recently, reports have emerged linking the use of oral NSAIDs and colchicine to impaired corneal wound healing and corneal perforation. This case report presents an instance of corneal melting and subsequent perforation in a medically unburdened patient who had been self-administering oral NSAIDs for one year. The evidence presented in this report suggests a plausible association between the prolonged administration of oral NSAIDs and corneal melt. Consequently, healthcare practitioners should be mindful of this potential risk when considering the prolonged use of oral NSAIDs.

## Introduction

Corneal melt and perforation can arise from a variety of causes, including infectious keratitis, collagen vascular diseases, trauma, and the use of toxic topical drops. Among the group of toxic topical drops known to impair corneal wound healing and lead to the development of corneal ulcers and perforation are topical non-steroidal anti-inflammatory drugs (NSAIDs), trifluridine, medications containing benzalkonium chloride (BAK), topical anesthetics, and topical beta-blockers. The literature extensively documents the association between the use of topical NSAIDs and the subsequent development of corneal ulcers [1-5]. More recently, reports have emerged indicating that the use of oral NSAIDs and colchicine can also impede corneal wound healing and lead to corneal perforation. This article aims to present a potential link between the use of oral NSAIDs and the occurrence of corneal melt followed by perforation in a medically uncomplicated patient.

This article was previously presented as an electronic poster at the 2022 ESCRS annual meeting in September 2022.

## Case presentation

A 79-year-old man, presented to our emergency room complaining of tearing and irritation for the past 15 days, associated with redness. This eye had been blind for a long time due to total cupping from undiagnosed advanced glaucoma. The patient denied any medical problems but reports using self-prescribed oral diclofenac 100 mg once daily for one year. He also denied a history of trauma or contact lens use. The systemic review was unremarkable.

On examination, visual acuity was poor light perception in the right eye and 20/40 in the left eye.

**Figure 1:**
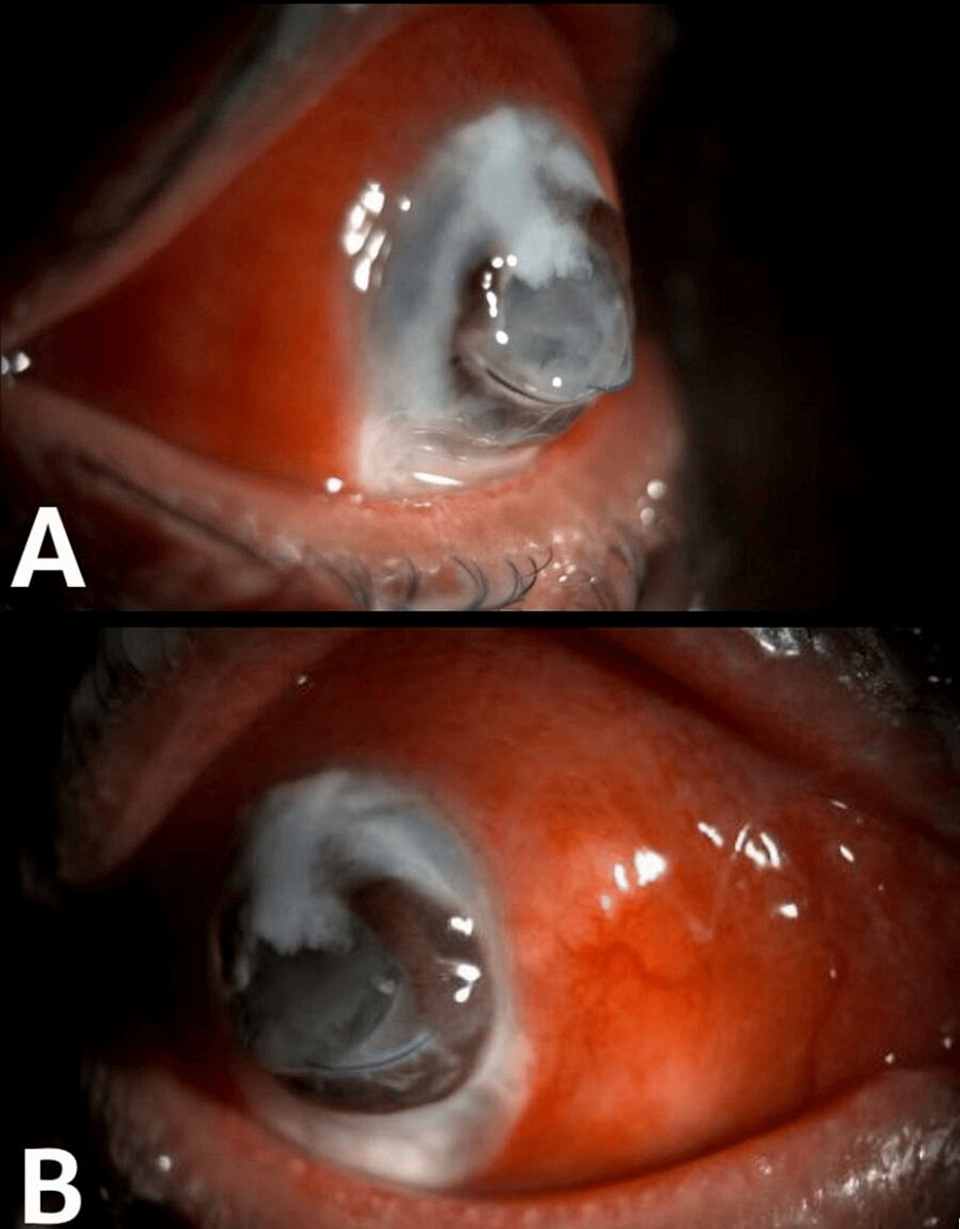
(A&B) Slit lamp photos of the patient at presentation showing corneal melt, globe perforation, and exposed IOL

Slit lamp examination of the right eye revealed swollen upper lid, 360 degrees injected conjunctiva with thick whitish discharge, complete corneal melt (almost 9x9 mm), intraocular lens (IOL) protruding with its haptics centrally, distorted iris, and no view to the fundus. The left eye revealed anterior blepharitis, clear and quiet conjunctiva, and sclera. Corneal thinning was noted superiorly from 11 to 12 peripherally with clear cornea and no leak. The rest of the slit lamp examination was unremarkable. The patient was found to have a large corneal perforation with exposed IOL secondary to corneal melt in the right eye (Figures 1A, 1B).

**Figure 2:**
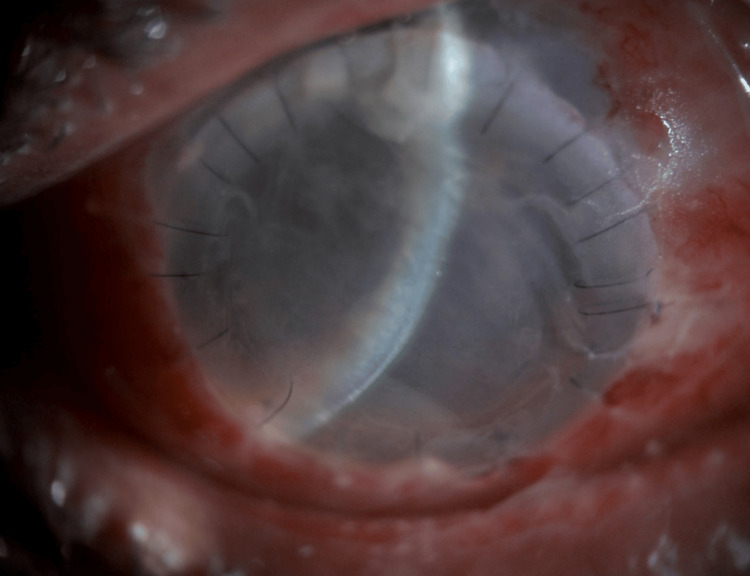
Slit lamp photos after management with tectonic penetrating keratoplasty

Options of evisceration versus tectonic penetrating keratoplasty were discussed with the patient and the decision was to undergo tectonic penetrating keratoplasty. The patient was admitted to the hospital and underwent emergent tectonic penetrating keratoplasty to salvage the eye given his poor visual potential (Figure 2).

Postoperatively, the pain gradually subsided. He was started on systemic broad-spectrum antibiotics to prevent infection. Corneal scrapping was done with culture and sensitivity and no organisms were seen. Excised tissue was sent to histopathology and showed disturbed iris tissue with mixed inflammation (predominantly chronic) with no organism seen.

Additional workup to identify the causes of corneal melt was done, which included complete blood count (CBC), erythrocyte sedimentation rate (ESR), and urgent imaging, which included serial B scan, computed tomography (CT) of the brain and orbit, and magnetic resonance imaging (MRI) of the brain and orbit, were performed.

The patient was also followed postoperatively. Almost all lab work was negative except for ESR, which was borderline high. Upon imagining, the patient was found to have choroidal detachment, a well-known finding in the presence of a long-standing leak.

Following a period of stable recovery, the patient was discharged and scheduled for regular follow-up appointments in the clinic. This comprehensive approach to the patient's care underscored the importance of ongoing monitoring and thorough investigation in cases of corneal melt and perforation associated with prolonged oral NSAID use.

## Discussion

This report describes a case of corneal melting and subsequent perforation in a medically free patient using a self-prescribed oral NSAID. Our patient presented with corneal perforation and exposed IOL haptics with fast progression, there was no history of trauma or contact lens use, he was not using any toxic topical eyedrops, and the results of culture and sensitivity were negative on repeated testing. Extensive investigations were done to exclude collagen vascular diseases, autoimmune conditions, immunosuppression, infectious causes, and chronic diseases like diabetes mellitus, chronic liver diseases, and renal failure came back negative. The only positive history was the use of oral NSAID, specifically, diclofenac 100 mg once daily for one year.

Ikuya Masuda et al. reported two cases of corneal perforation after oral NSAID use for five months and seven days, respectively [6]. Alster et al. discussed two cases of delayed corneal wound healing after the use of oral colchicine [7]. Topical NSAID can induce corneal melt by causing corneal epithelial defect, reduced eicosanoid levels, leukocyte infiltration, and matrix metalloproteinase-facilitated desquamation and stromal degradation [8]. Many oral medications used in the treatment of corneal disease can reach the anterior segment and posterior segment through the blood-aqueous barrier and blood-retinal barrier [9]. The hypothesized mechanism of action of oral NSAIDs is by inhibiting cyclooxygenases (COXs) and the COX product 12(S)-hydroxyheptadeca-5Z,8E,10E-trienoic acid (12-HHT), which was found as an endogenous ligand for leukotriene B4 receptor 2 (BLT2), which has a great role in maintaining the corneal epithelial homeostasis [10].

We understand that other risk factors can lead to this, but we are certain that the patient’s use of diclofenac 100 mg once daily for that long duration led to the delay in presentation.

The patient was blind in this eye and was self-medicating with the use of NSAID; therefore, this case reports an extreme presentation of corneal perforation with exposed eye contents (including iris and IOL), we think that this delay is secondary to a lack of alarming signs such as decrease of vision in an initially poor seeing eye and pain due to NSAID-induced decreased corneal sensation.

## Conclusions

This case report emphasizes the possible effects of long-term oral NSAID use and the necessity for healthcare providers to be more knowledgeable of the potential ocular side effects of these medications.
